# A comprehensive scoping review of tibial cysts after anterior cruciate ligament reconstruction

**DOI:** 10.1186/s40634-021-00356-9

**Published:** 2021-06-21

**Authors:** Nuno Camelo Barbosa, João Pedro Campos, Vânia Capelão, Vikram Kandhari, Thais Dutra Vieira, Bertrand Sonnery-Cottet

**Affiliations:** 1 Hospital Pedro Hispano, Hospital Pedro Hispano Rua Dr. Eduardo Torres, Matosinhos, Portugal; 2 Centro Hospitalar Lisboa Norte, Lisboa, Portugal; 3 Sydney Orthopaedic Research Institute, Sydney, Australia; 4 Centre Orthopédique Santy, Hopital Privé Jean Mermoz, Ramsay-Générale de Santé, Lyon, France

**Keywords:** ACL, Tibial cyst, Pretibial cyst, Interference screw

## Abstract

**Purpose:**

The purpose of this study was to perform a scoping review of published literature reporting on surgical management of tibial cysts which developed after ACLR.

**Methods:**

A scoping review was conducted following the Arksey and O’Malley framework for scoping studies and Preferred Reporting Items for Systematic Reviews and Meta-analyses (PRISMA) extension for scoping reviews (PRISMA-ScR) guidelines. A search strategy using the terms [“Tibial Cyst” AND “ACL”], [“Pretibial Cyst” AND “ACL”] was applied to the PUBMED database.

**Results:**

Thirty-seven studies published between 1990 and 2019 were a part of this scoping review. Non-absorbable implants for tibial graft fixation were used in 10 studies (comprising a total 21 patients), while bio-absorbable implants were used in 27 studies (comprising a total 115 patients). Incidence of tibial cyst was reported in 3 studies (434 primary ACLRs) from whom 3.9% (*n* = 17) developed tibial cyst. Tibial cyst development in relation to use of bio-absorbable screws for tibial ACL graft fixation was reported in 16 studies (42.1%). Use of bio-absorbable screws with another factor was found to be related to tibial cyst development in another 1 study (2.6%). Most common symptoms were presence of mass or swelling, pain, tenderness, drainage, instability and effusion.

**Conclusion:**

This scoping review demonstrated that tibial cysts is more frequently related to bioabsorbable screws, however it can also occur due to other causes. Current literature on tibial cyst after ACLR is of low-quality evidence. Future research is required to better understand aetiology, risk factors for cyst formation and the best possible mode of management.

**Level of evidence:**

IV

**Supplementary Information:**

The online version contains supplementary material available at 10.1186/s40634-021-00356-9.

## Background

Anterior Cruciate Ligament Reconstruction (ACLR) has been associated with significantly improved patient reported outcomes with respect to quality of life, knee symptoms and sports function when compared to non-operative treatment for patients with anterior cruciate ligament (ACL) tears [5]. Development of tibial cyst following ACLR is a rare but known complication of ACLR. To our knowledge Sgaglione was the first to report a tibial cyst related to ACLR [57].

Tibial graft fixation in ACLR was initially attained with staples, screws, washer posts and sutures tied directly to bone. Significant improvements have been witnessed in the make and design of implants for tibial graft fixation. Bio-absorbable screws have been developed and their use has facilitated surgeons to overcome some complications related to non-absorbable implants [50]. Bio-absorbable materials are a popular method of tibial fixation due to advantages like the absence of artefacts on postoperative magnetic resonance imaging (MRI), simpler revision surgery and less graft damage compared to metallic implants [2, 16, 30, 44]. Unfortunately, bio-absorbable screws aren't exempt of complications, and several authors have related them to tibial cyst development after ACLR and ghost screws formation [16, 25, 53].

Furthermore, available literature about surgical treatment of tibial cysts following.

ACLR is scarce. For these reasons, a scoping review, was conducted in order to map the extent, range and quality of literature associated with development of tibial cysts after ACLR, giving an overview that further helps clinicians. A scoping review methodology was selected because this approach is considered to be superior when addressing an exploratory research question [27, 47].

## Review

### Study selection

A scoping review of the literature was conducted in accordance with the Preferred Reporting Items for Systematic Reviews and Meta-analyses (PRISMA) extension for scoping reviews (PRISMA-ScR) guidelines [63] and the methodological framework of Arksey and O´Malley [6]. The study protocol was registered with the open science framework study registry prior to commencing data collection – OSF [73] database (reference blinded for review). The five-stage methodological framework in a scoping review of Arksey and O’Malley [6] were followed: as (1) the identification of a research question; (2) identifying the relevant studies; (3) the selection of studies to be included in the review; (4) data extraction from the included studies; and (5) collating, summarizing, and reporting the results of the review.

1. **Identification of research questions**
  The research question was “What is known from the existing literature regarding development and management of tibial cyst after ACLR?”.
2. **Identifying Relevant Studies**
  Studies were identified by applying the search strategy to the PubMed database. The following keywords were included [“Tibial Cyst” AND “Anterior Cruciate Ligament”], [“Pretibial Cyst” AND Anterior Cruciate Ligament”] with automatic mapping to Medical Subject Headings terms. The search was conducted on May 16, 2020 (search date last executed), by 2 independent investigators (XX. and YY) (Table 1). Limits were applied to retrieve English-language, Spanish-Language and Portuguese-Language articles published. Both investigators reviewed the titles and abstracts of all identified records and potentially eligible studies were retrieved for full-text review. Reference lists of these articles were also reviewed, and any further potentially eligible studies were identified.
3. **Study selection**
  All identified studies reporting clinical outcomes of tibial cyst surgery after ACLR were included. The following article types were excluded: non-clinical studies such as cadaveric and animal studies. The senior author resolved any disagreements between investigators regarding whether a study met the eligibility criteria.
4. **Data Extraction**

**Table 1:** Literature search sequence on Pubmed—Tibial cysts after ACLR (last performed on March 27, 2020)

1	Tibial Cyst ACL	94 items
2	Tibial Cyst	1218 items
3	Pretibial cyst ACL	24 items
4	Pretibial Cyst	32 items

The included studies were analysed in details and data from each was recorded in Excel 2013 (Microsoft Corp., Redmond, WA) and then subjected to a stepwise analysis. The recorded data from each study included patients’ demographic and clinical information, imaging findings and peri-operative findings. With demographics, patients’ clinical information consisted of the symptoms at presentation, their duration and their effect on activities of daily living. The imaging findings recorded from the pre-operative MRI were presence of tibial tunnel enlargement and presence of tibial communication with the knee joint. Recorded peri-operative findings included details of surgical technique for managing tibial cyst, status of bio-absorbable screws, and intra operative testing of joint communication with tibial cyst. Findings of tissue sample screening by a microbiologist, and histopathologist were recorded. Complications including failure (defined as recurrence of tibial cyst after surgical excision) were recorded and evaluated.

### Collating, summarizing and reporting the results

Due to a small number of published studies and heterogeneity between them, no statistical analyses were performed. Instead, the findings were summarized through a narrative analysis of the included published literature. The risk of bias in included case series was assessed using the Methodological Index for Non-Randomized Studies (MINORS) [60]. Overall quality of evidence for each of the potential risk factors studied was assessed using the GRADE (Grading of Recommendations Assessment, Development and Evaluation) working group criteria [28].

## Results

**Fig. 1:**
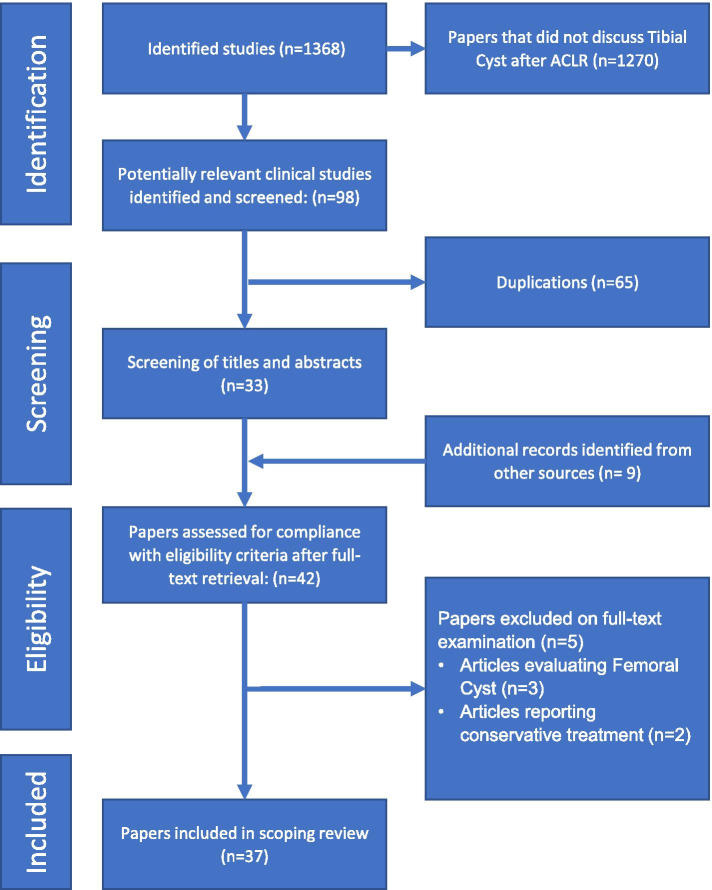
Flow-chart of identification, screening, and selection of studies

Application of the search strategy identified 1368 records from the searched databases. With title and abstract screening, 98 potentially relevant studies were isolated. 65 studies were removed as they were duplicates, 9 additional records identified from another source (33 studies references review) and 5 papers were excluded on full text examination. Thirty-seven studies were eligible for inclusion in the systematic review. The flow-chart of studies is represented in Fig. 1. The publication dates of the included studies ranged from 1999 to 2019. Using the adjusted Oxford Center For Evidence-Based Medicine criteria [74, 75] for the level of evidence we found that 1 study was Level I [11], 1 Level II [25], 1 Level III [57] and 34 studies were level IV [1, 3, 10, 12–17, 19–21, 23, 29, 33, 36, 42, 43, 46, 49, 51, 53, 54, 56, 58, 59, 62, 64–66, 69, 71, 72] case series or case reports.

### Basic characteristics of included studies

From all the included studies, 136 patients were evaluated with mean age of 31.0 (14 – 57) years. Main symptoms following tibial cyst development after ACLR were mass or swelling in the area of tibial tunnel, pain, instability, and fluid discharge from the earlier surgical incision. Mean time to surgery was 40.2 (0.2 – 240) months. Incidence was calculated in 3 studies comprising a total 434 patients from whom 3.9% (*n* = 17) developed tibial cyst. Follow-up after the surgery for tibial cysts was reported in 28 studies comprising a total 122 patients. The mean duration of reported follow-up was 37.7 (2 – 70) months. All the data of interest from the included studies have been illustrated in Table 2.

**Table 2:** Demographic, radiological and surgical data from included studies

*Authors*	*Date*	*Patients*	*Age*	*ACL surgery*	*Fixation method*		*Bioabsorbable interference screw*
*Sgaglione NA*	1990	1 (NA)	NA	repair +—semitendinous augmentation	NR		
*Brettler D*	1995	1 (M)	41	autograft bptb	metal interference screw		
*Victoroff BN*	1995	4 (3 M)	31 (17–47)	allograft achilles	staples / screw washer		
*Simonian PT*	1998	3 (M)	29,3 (25–35)	2 autograft hamstring 1 autograft iliotibial band	2 staple; 1 in situ		
*Martinek V*	1999	1 (M)	32	autograft bptb	PDLLA interference screw		Sysorb, Sulzer Orthopedics
*Deie M*	2000	2 (F)	26,3 (20, 33)	autograft hamstring	2 staples		
*Brager MA*	2002	3 (2 M)	39,7 (36–47)	autograft hamstring	1 screw washer 2 in situ		
*Malhan K*	2002	1 (F)	22	autograft hamstring	PLLA + β-TCP interference screw		BioLok, Atlantech
*Ilahi OA*	2003	1 (F)	26	autograft bptb	metal interference screw		
*Sekiya JK*	2004	1 (F)	16	autograft hamstring	over a post Ethibond		
*Tsuda E*	2006	1 (M)	18	autograft hamstring	PLLA interference screw + over a post Ethibond		Fixsorb, Depuy Mitek
*Busfield BT*	2007	2 (F)	34 (28, 39)	1 allograft achilles 1 autograft hamstring	1 PLLA interference screw + screw washer; 1 PLLA interference screw + over a post		1 PLLA Delta, Arthrex 1 Bioscrew, Arthrex
*Thaunat M*	2007	1 (F)	47	autograft hamstring	PLLA interference screw		Phusiline, Phusis
*Dujardin J*	2008	1 (M)	32	autograft hamstring	PDLG + CA interference screw		Calaxo, Smith and Nephew
*Gaweda K*	2009	2 (NA)	NA	autograft hamstring	PLLA interference screw		NR, Arthrex
*Umar M*	2009	1 (M)	28	autograft hamstring	PLLA + β-TCP interference screw		BiLok, Arthocare
*Sadat-Ali M*	2010	1 (M)	23	autograft hamstring	PLLA interference screw		NR, Bionix Implants
*Oh HL*	2010	1 (F)	15	autograft hamstring	PLLA + β-TCP interference screw		Bio-INTRAFIX, Depuy Mitek
*Gonzalez-Lomas G*	2011	7 (NA)	39,3 (22–57)	4 autograft hamstring 3 allograft	PLLA interference screw		PLLA Delta, Arthrex
*Quatman CE*	2011	1 (F)	28	allograft tibialis anterior	PLLA + HA interference screw + staple		BioRCI, Smith and Nephew
*Apostolopoulos A*	2012	1 (M)	26	autograft hamstring	PLLA interference screw		NR
*Bernard JA*	2013	3 (2 M)	28 (25–32)	2 autograft hamstring 1 allograft tibialis anterior	2 PDLLA + β-TCP interference screw + PLLA SwiveLock 1 PLLA interference screw		2 BioComposite, Arthrex 1 Bio-Interference, Arthrex
*Bourke HE*	2013	1 (NA)	NA	autograft hamstring	PDLG + CA interference screw		Calaxo, Smith and Nephew
*Shen MX*	2013	1 (M)	21	autograft hamstring	PLLA interference screw		Bio-Interference, Arthrex
*Bulisani LEP*	2014	1 (M)	40	autograft hamstring	PLLA + HA interference screw		NR
*Díaz Heredia J*	2014	3 (1 M)	25 (23–27)	autograft hamstring	PLLA + HA interference screw		Biosure, Smith and Nephew
*Ramsingh V*	2014	14 (9 M)	27,1 (14–39)	12 autograft hamstring 1 autograft bptb 1 allograft achilles	PLLA + β-TCP interference screw		BiLok, Arthocare
*Zabala IL*	2014	1 (M)	29	autograft hamstring	PLLA interference screw		NR
*Haragus H*	2015	1 (M)	20	autograft hamstring	PLLA interference screw		NR
*Joshi YV*	2015	1 (M)	27	autograft bptb	metal interference screw		
*Metcalf K*	2015	2 (1 M)	39,5 (25–54)	allograft tibialis anterior	PDLLA + β-TCP interference screw		Bio-INTRAFIX, Depuy Mitek
*Alonso B*	2016	1 (M)	17	autograft bptb	PDLLA + β-TCP interference screw		Megafix C, Karl Storz
*Zicaro JP*	2017	13 (9 M)	35,6 (NA)	autograft hamstring	6 bioabsorbable interference screw 3 bioabsorbable interference screw + staple 4 in situ + Ethibond		NR
*Weiss KS*	2017	1 (F)	20	autograft hamstring	PDLLA + β-TCP interference screw		ComposiTCP; Biomet
*Christodoulidis A*	2018	2 (1 M)	50,5 (49, 52)	autograft hamstring	PLLA interference screw + PLLA cross pin		Bio-Interference, Arthrex
*Chevallier R*	2019	53 (20 M)	30,8 (NA)	44 autograft hamstring 8 autograft bptb 1 autograft iliotibial band	28 PLLA + HA 10 PLLA + β-TCP 7 PLLA 5 bioabsorbable interference screw 2 PDLG + CA 1 PLGA		21 BioRCI, Smith and Nephew 7 Biosure, Smith and Nephew 6 Bio-INTRAFIX, Depuy Mitek 3 Milagro, Depuy Synthes 1 TLS, FH orthopedics 7 GTS, Smith and Nephew 5 NR 2 Calaxo, Smith and Nephew 1 CentraLoc, Biomet
*Dockry A*	2019	1 (M)	33	allograft NR	bioabsorbable interference screw		NR
***Authors***	**Time to diagnosis/surgery**		**Presentation**	**Tunnel enlargment**	**MRI joint communication**		**Surgery joint communication**
*Sgaglione NA*	44		NR	NR	NR		no
*Brettler D*	22		mass tenderness	yes	NR		yes
*Victoroff BN*	15,8 (7–29)		2 mass 2 mass tenderness	NR	NR		3 yes 1 NR
*Simonian PT*	46,3 (25–72)		mass tenderness	no	1 yes 2 NR		yes
*Martinek V*	8		mass tenderness	yes	no		NR
*Deie M*	16 (15–17)		Mass	yes	NR		no
*Brager MA*	46,3 (21–76)		mass tenderness	1 yes 2 NR	1 yes 2 NR		1 yes 1 no 1 NR
*Malhan K*	12		mass tenderness	yes	no		NR
*Ilahi OA*	12		mass tenderness	NR	NR		yes
*Sekiya JK*	60		mass tenderness	yes	no		no
*Tsuda E*	24		mass tenderness	yes	yes		yes
*Busfield BT*	27 (18–36)		mass tenderness	NR	NR		no
*Thaunat M*	60		mass tenderness	yes	no		NR
*Dujardin J*	8		effusion	yes	no		no
*Gaweda K*	18 (16–20)		1 drainage 1 mass tenderness	NR	NR		NR
*Umar M*	30		mass tenderness	NR	NR		no
*Sadat-Ali M*	36		mass tenderness	no	NR		no
*Oh HL*	4		drainage	NR	no		no
*Gonzalez-Lomas G*	29,1 (24–36)		4 mass 2 mass tenderness 1 drainage	yes	4 yes 3 no		NR
*Quatman CE*	78		mass tenderness	yes	yes		no
*Apostolopoulos A*	48		Mass	yes	NR		no
*Bernard JA*	20,7 (16–24)		1 mass 2 mass tenderness	NR	NR		NR
*Bourke HE*	9		Mass	NR	no		NR
*Shen MX*	24		mass tenderness	no	no		no
*Bulisani LEP*	36		Mass	no	yes		yes
*Díaz Heredia J*	0,3 (0,2–0,5)		drainage	no	yes		2 yes 1 no
*Ramsingh V*	27,8 (11,5–38,5)		12 mass tenderness 2 mass tenderness effusion	NR	NR		2 yes 12 no
*Zabala IL*	24		mass tenderness	yes	no		no
*Haragus H*	42		mass tenderness	yes	no		no
*Joshi YV*	240		instability	no	no		no
*Metcalf K*	22 (8–36)		mass tenderness	no	1 no 1 NR		no
*Alonso B*	24		mass tenderness	NR	NR		no
*Zicaro JP*	29 (NA)		Mass	yes	no		NR
*Weiss KS*	15		Mass	no	no		no
*Christodoulidis A*	84 (84–84)		1 mass; 1 mass tenderness	no	no		no
*Chevallier R*	55,2 (3,1–228)		22 mass tenderness 31 tenderness	4 yes 49 NR	2 yes 51 no		4 yes 49 no
*Dockry A*	16		mass tenderness	NR	NR		NR
***Authors***	**Surgery**		**Screw degradation**	**Microbiology**	**Histology**	**Follow-up (M)**	**Recorrence**
*Sgaglione NA*	excision		NR	NR	NR	NR	NR
*Brettler D*	excision		NR	NR	NR	18	no
*Victoroff BN*	1 excision + curetage 3 excision + curetage + bone graft		NR	NR	1 no 3 yes	24,8 (22–29)	1
*Simonian PT*	1 excision + curetage; 2 excision + curetage + bone graft		NR	NR	1 no 2 yes	39,3 (24–70)	no
*Martinek V*	excision		breakdown	NR	yes	2	no
*Deie M*	excision		NR	NR	yes	10,5 (9–12)	no
*Brager MA*	2 excision + curetage 1 excision + curetage + bone graft		NR	NR	yes	30 (6–54)	no
*Malhan K*	excision + curetage		breakdown	negative	yes	3	no
*Ilahi OA*	excision + curetage + bone graft		intact	NR	yes	2	no
*Sekiya JK*	excision + curetage		NR	NR	no	NR	1
*Tsuda E*	excision + curetage + bone graft		breakdown	negative	yes	12	no
*Busfield BT*	excision + curetage		breakdown	negative	yes	6	no
*Thaunat M*	excision + curetage + bone graft		absorption	NR	yes	2	no
*Dujardin J*	excision + curetage + bone graft		breakdown	negative	yes	3	no
*Gaweda K*	excision + curetage		intact	NR	yes	NR	no
*Umar M*	excision + curetage		breakdown	NR	yes	NR	no
*Sadat-Ali M*	excision + curetage		breakdown	NR	yes	9	no
*Oh HL*	excision + curetage		intact	M. fortuitum	no	NR	no
*Gonzalez-Lomas G*	excision		NR	negative	yes	5,3 (5–6)	no
*Quatman CE*	excision + curetage + bone graft		NR	NR	no	NR	no
*Apostolopoulos A*	excision + curetage + bone graft		absorption	NR	yes	12	no
*Bernard JA*	2 excision 1 excision + curetage		breakdown	negative	no	4 (2–6)	no
*Bourke HE*	excision		NR	NR	no	NR	1
*Shen MX*	excision		absorption	negative	yes	NR	no
*Bulisani LEP*	excision + curetage + bone graft		absorption	NR	yes	6	no
*Díaz Heredia J*	excision + curetage		intact	negative	yes	24	no
*Ramsingh V*	excision + curetage		13 breakdown 11 absorption	negative	yes	12	no
*Zabala IL*	excision + curetage		breakdown	NR	yes	3	no
*Haragus H*	excision		intact	NR	yes	3	no
*Joshi YV*	excision + curetage + bone graft		NR	NR	yes	6	no
*Metcalf K*	excision + curetage		1 intact 1 breakdown	P. Acnes none	yes	12	no
*Alonso B*	excision + curetage		breakdown	negative	yes	2	no
*Zicaro JP*	6 excision + curetage 7 excision + curetage + bone graft		NR	negative	yes	35 (NR)	1
*Weiss KS*	excision + curetage + bone graft		absorption	S. epidermidis	yes	4	no
*Christodoulidis A*	excision + curetage		absorption	NR	yes	6, NR	no
*Chevallier R*	excision + curetage + bone graft		12 intact 32 breakdown 9 absorption	negative	12 no 41 yes	64,8 (7–146)	1
*Dockry A*	excision + curetage + bone graft		NR	negative	no	NR	no

Non-absorbable implants for tibial graft fixation were used in 10 studies (comprising a total 21 patients), while bio-absorbable implants were used in 27 studies (comprising a total 115 patients). Composition of bio-absorbable screws and frequency of development of tibial ACL cysts with their use are described in Table 3.

**Table 3:** Method of fixation, interference screw composition and auxiliar fixation frequency

***Fixation method***	*Frequency*
*NR*	1 (0,7%)
*None*	3 (2,2%)
*Screw washer*	1 (0,7%)
*Screw washer* + *metal interference screw*	1 (0,7%)
*Screw washer* + *staple (removed before cyst development)*	1 (0,7%)
*Screw washer* + *2 staples (removed before cyst development)*	1 (0,7%)
*Screw washer* + *poly-L-lactide (PLLA) interference screw*	1 (0,7%)
*Staple*	2 (1,5%)
*Staples 2*	3 (2,2%)
*Staples 2 (removed before cyst development)*	1 (0,7%)
*Staple* + *not reported bioabsorbable interference screw*	3 (2,2%)
*Staple* + *poly-L-lactide (PLLA)* + *hydroxyapatite (HA) interference screw*	1 (0,7%)
*Ethibond*	4 (2,9%)
*Over a post Ethibond*	1 (0,7%)
*Over a post Ethibond* + *poly-L-lactide (PLLA) interference screw*	1 (0,7%)
*Over a post* + *poly-L-lactide (PLLA) interference screw*	1 (0,7%)
*Bioabsorbable cross pin in PLLA* + *poly-L-lactide (PLLA) interference screw*	2 (1,5%)
*PLLA SwiveLock* + *poly-D,L-lactide (PDLLA)* + *β-tricalcium phosphate (β-TCP) Interference screw*	2 (1,5%)
*Metal interference screw*	2 (1,5%)
*Not reported bioabsorbable interference screw*	12 (8,8%)
*Poly(lactic-co-glycolic) acid (PLGA) interference screw*	1 (0,7%)
*Poly-L-lactide (PLLA)* + *hydroxyapatite (HA) interference screw*	32 (23,5%)
*Poly-L-lactide (PLLA)* + *β-tricalcium phosphate (β-TCP) interference screw*	27 (19,9%)
*Poly-L-lactide (PLLA) interference screw*	23 (16,9%)
*Poly-D,L-lactide (PDLLA)* + *β-tricalcium phosphate (β-TCP) interference screw*	4 (2,9%)
*Poly-D,L-lactide (PDLLA) interference screw*	1 (0,7%)
*Poly(lactic-co-glycolic) acid (PLGA) interference screw*	1 (0,7%)
*Poly(D,L-lactideecoglycolide) (PDLG)* + *calcium carbonate interference screw*	4 (2,9%)
***Interference screw composition***	Frequency
*NR*	1 (0,7%)
*None*	17 (12,5%)
*Metal*	3 (2,2%)
*Not reported bioabsorbable*	15 (11%)
*Poly-L-lactide (PLLA)*	28 (20,6%)
*Poly-L-lactide (PLLA)* + *hydroxyapatite (HA)*	33 (24,3%)
*Poly-L-lactide (PLLA)* + *β-tricalcium phosphate (β-TCP)*	27 (19,9%)
*Poly-D,L-lactide (PDLLA)*	1 (0,7%)
*Poly-D,L-lactide (PDLLA)* + *β-tricalcium phosphate (β-TCP)*	6 (4,4%)
*Poly(lactic-co-glycolic) acid (PLGA)*	1 (0,7%)
*Poly(D,L-lactideecoglycolide) (PDLG)* + *calcium carbonate*	4 (2,9%)
***Auxiliar fixation***	Frequency
*NR*	1 (0,7%)
*None*	109 (80,1%)
*Removed before cyst*	3 (2,2%)
*Screw washer*	3 (2,2%)
*Staple*	6 (4,4%)
*Staples 2*	3 (2,2%)
*Ethibond*	4 (2,9%)
*over a post Ethibond*	2 (1,5%)
*over a post*	1 (0,7%)
*Bioabsorbable cross pin in PLLA*	2 (1,5%)
*PLLA SwiveLock*	2 (1,5%)

The methodological quality of included case series evaluated by the MINORS tool varied between 5 and 8 indicating a high risk of bias (Additional file 1).

 The overall strength of the evidence available in the scoping review using GRADE recommendations (Table 4) was very low.

**Table 4:** Quality of evidence of literature on Tibial cyst development after ACLR

Risk Factor	Risk of Bias	Inconsistency	Indirectness	Imprecision	Grade
Bioabsorbable screw	likely	unexplained heterogeneity	indirect	imprecision	very low
Tibial Comunication	unlikely	unexplained heterogeneity	indirect	imprecision	very low
Graft Type	unlikely	unexplained heterogeneity	indirect	imprecision	very low
Infection	unlikely	unexplained heterogeneity	indirect	imprecision	very low

### Tibial cyst development

Tibial cyst development in relation to use of bio-absorbable screws for tibial ACL graft fixation was reported in 16 studies (42.1%). Use of bio-absorbable screws and reaction to suture material was found to be related to tibial cyst development in one study (2.6%) [64]. Development of tibial cyst was also related to communication between the tibial tunnel and knee joint in 8 studies (21.1%), other causes were appointed in 9 articles (21.1%): increased synovial fluid production [13], tendon necrosis [19], suture fragments reaction [56], allograft tendon [10], graft micro-motion [36], infection [46, 49, 69] and multifactorial aetiology [72]. Also, 3 studies did not provide any information on the reason for development of tibial cysts.

### Imaging findings

Tibial tunnel enlargement was assessed in 25 studies comprising of 53 patients. Thirty-eight (71.7%) of them were found to have ACL tibial tunnel enlargement in either pre-operative x-ray or MRI scan done before the surgery for tibial cyst.

Communication of the ACL tibial tunnel with the knee joint was evaluated in preoperative MRI scans in 23 studies (comprising a total 91 patients).

Communication could be identified in 14 patients and was not present in 85.4% (*n* = 80) patients.

### Surgical findings

Surgical procedure technique was reported in 37 articles (comprising a total 136 patients) in 55.9% (*n* = 76) of them, cyst excision was associated with curettage and bone (allo or auto) grafting. Also, in 12,5% (*n* = 17) isolated cyst excision was performed and in 31.6% (*n* = 43) curettage and excision were performed.

Screw absorption status at time of surgery was reported in 24 articles comprising a total 97 patients, 21.6% (*n* = 21) of them reported an intact screw implant, 60.8% (*n* = 59) presented a partially resorbed screw and in 17.9% (*n* = 17) screw was completely resorbed at the time of tibial cyst surgery.

In 90% of patients autograft was used (*n* = 122, 106 hamstring, 14 patellar tendon, 2 iliotibial band). The remaining used allograft (n = 14, 6 Achilles tendon, 4 tibialis anterior tendon, 4 NR).

### Tissue processing

Samples from the cyst were sent for processing either to the microbiologist and/or to the histopathologist. Presence of infection was reported in 3 patients from 16 studies (comprising a total 102 patients) in which the tissue sample was sent to the microbiologist for evaluation. Organism isolated in these 3 patients was different in each. *Staphylococcus epidermidis*, *Propionibacterium acnes and Mycobacterium fortuitum* were the organisms isolated in the three patients.

Tissue sample was sent for analyses to a histopathologist in 29 studies, comprising a total 112 patients. Foreign body reaction was found to be present in 10 patients (9%).

### Complications

The only reported complications of Tibial cyst excision after ACLR were recurrences of tibial cyst after surgical management reported in 4 patients in 4 different studies.

## Discussion

The most important finding of this study is that tibial cyst in ACLR, is more frequently related to bio-absorbable implants, however it also has been related to other causes.

### Clinical presentation and aetiology

Our scope identified tibial cysts occurring with several types of fixation methods, screw composition and auxiliar fixation methods as described in Table 3. Typically, tibial cyst after ACLR presents with mass or tenderness over the distal tibial aperture within 40.2 months after the primary procedure, although immediate or late-term presentations have also been reported.

This scoping review reveals that tibial cyst development after ACLR is a rather uncommon condition. Incidence of tibial cysts was reported by Ramsingh et al. being up to 5% at 2–3 years [53]. Overall in this review, incidence could be calculated in 3 articles totalling a total 434 patients, 3.9% of them (n = 17) developed tibial cyst after ACLR.

Bioabsorbable implants were used in 27 studies and non-absorbable implants for tibial graft fixation were used in 10 studies. The biggest frequencies of tibial cysts were associated to bioabsorbable screws – 23.5% were poly(L-lactic) acid (PLLA) + hydroxyapatite (HA), 19.9% were PLLA + B-tricalcium phosphate (B-TCP) and 16.9% were PLLA interference screws (Table 3).

Tibial cyst formation has been linked to several causes, such has foreign body reaction [53], leakage of joint fluid through the tunnel [62], intraosseous graft necrosis with incomplete graft incorporation [66] and graft micro-motion [59, 64, 66], among other causes. Development of tibial ACL cysts has also been controversially linked to the tibial graft fixation methods. [26, 59, 62, 64, 66]. In our scoping review, almost half (42.1%) of the studies related tibial cyst development to the use of bio-absorbable implants.

### Bio-absorbable implants

Bio-absorbable implants were developed in order to address the limitations with the use of non-absorbable implant. Some of the concerns with the use of non-absorbable implants include screw breakage, artefact in MRI, and hardware interference in ACL Revision and subsequent need for hardware removal [44]. The natural history of the bio-absorbable implant is that it will be absorbed and replaced by bone in the tibial tunnel, however this isn´t consistently seen in vivo [7, 52, 67]. Through our review we found complete absorption of the screw evident in only 17 (17.9%) patients. Others either remain partially resorbed or un-resorbed. Also, though bio-absorbable address some of the limitations encountered with the use of non-bioabsorbable screws, their use is not without complications. Complications in ACLR, [38] related to the use of bio-absorbable tibial ACL screws include foreign body reaction [26], breakdown [64], migration and tibial cyst formation.

Degradation of bioabsorbable materials occurs over five stages: hydration, depolymerization, loss of mass integrity, absorption and elimination [52]. During hydrolysis, the screw may release acid products (resultant from screw composition degradation) harmful to surrounding tissues. As so, different materials result in different degradation products, with different effects on surrounding tissues, and different timings of degradation which may lead to fluid collection on the bone tunnel and progress to tibial cysts [68, 70].

Bone tunnel fluid collections are common in ACLR, however not all fluid collections in the bone tunnel mature into tibial cysts [67]. Moreover, fluid collection can resolve [55]. Chevallier et al. present the biggest series of reported tibial cysts after ACLR in a retrospective clinical study that included 53 patients with an average 4.6 years (+ -3.1 months) after primary ACLR. The authors found that bio-absorbable interference screws absorption can be symptomatic independent of screw composition and correlated tibial cysts to bio-absorbable screw absorption [16]. Unfortunately, the authors didn´t provide individual results database.

However, some prospective imaging studies following up bio-absorbable implants fail to report on tibial cysts. Tecklenburg et al. despite a short follow-up of 24 months after ACLR, reported no inflammatory response in the tibial tunnels in a prospective imaging study of patients with bio-absorbable and allograft screws [61]. Furthermore, Barber et al. in a long-term study of bio-absorbable screws degradation, demonstrated no tibial cysts and complete degradation with no screw remnant at 3 years after BPTB (Bone patella tendon bone) graft ACLR in 14 patients [8]. Also, Jonhston et al. in a computed tomography study of 65 patients after ACLR with bioabsorbable screw showed no tunnel enlargement, osteolysis or reported tibial cysts at long term [35]. Thus, other causes may also be related to tibial cyst development.

### Non-absorbable implants and other tibial cyst causes

Tibial cysts development was already described in early ALCR articles with non-absorbable methods of fixation. Our scoping review included 10 articles in which nonabsorbable implants were used for graft fixation. These authors related tibial cysts with several causes such as drainage from the joint through the tibial tunnel, which could be caused by a tunnel with difference in diameter in relation to the graft, eccentric positioning of the tendon in the bone tunnel, intraosseous tendon necrosis during graft incorporation [19], incomplete allograft incorporation [15, 33, 59, 66], graft micro-motion [36, 59, 64, 66], synovitis [13] and foreign body reaction due to non-absorbable suture [56].

Victoroff et al. and Simonian et al. described tibial cyst after ACLR with non-absorbable implants, the authors associated incomplete graft tissue incorporation in the bone tunnel to tibial cysts. Accordingly, graft necrosis led to synovialization that allowed synovial fluid to be transmitted through the tibial tunnel [59, 66]. As so, hydrostatic pressure within the knee joint would drive synovial fluid allowing accumulation and development of tibial cyst [41, 64, 66].

Furthermore, prospective imaging studies have failed to show difference in tibial cyst formation between bio-absorbable and non-absorbable fixation implants.

In a systematic review by Debieux et al. [18] on bio-absorbable versus metallic screws for graft fixation in anterior cruciate ligament reconstruction, the authors chose to include 12 randomised controlled trial published between 1995 and 2015 [4, 9, 22, 24, 31, 32, 34, 37, 39, 40, 45, 48]. Of the included studies only Arama et al. reported tibial cyst formation, and according to the authors there were no differences between bio-absorbable (4 of 17 pts PLLA-HA) and non-bioabsorbable (3 of 19 pts Titanium) groups in cyst formation or graft integration [4].

### Surgical preference

In our scoping review surgical resection and bone grafting was the most preferred surgical approach in 84 patients (61.76%). Tibial cyst recurrence was reported in only 4 patients [11, 56, 66, 72].

### Communicating vs non-communicating tibial cyst

Distinguishing between communicating and non-communicating cysts might be helpful in further understanding the cause of tibial cyst development as described by Zicaro [72]. Communication between joint and tibial tunnel is in theory always possible after ACLR procedure. Depending on the amount of communication, hydrostatic pressure in the tibial tunnel may lead to tibial cyst formation at early, medium or long-term [66]. Thus more than one factor may be responsible for formation of tibial ACL cysts as pointed out by Zicaro [72] and other authors.

This review identified when using bio-absorbable implants (28 articles), 19 (67.8%) articles evaluated tibial communication with the joint with MRI and communication was found in 12 (13.6%) patients. During surgical procedure 19 (67.8%) articles evaluated tibial communication with the joint communication – it was found in 10 (11.2%) patients. However, probing the tibial tunnel with an arthroscopic probe may not be enough to rule out tibial tunnel communication. Noteworthy, in our review only one article performed a fistulogram with radiographic contrast dye in order to confirm communication of tibial tunnel with the joint [66].

### Histopathology

In our scope we found 10 patients with histopathology report of foreign body reaction, overall, we encountered great variability among the reports (Table 2).

### Study limitations and strengths

The limitation of this of this scoping review is the inclusion of mostly level IV studies. However, it is worthy to include them as the incidence of occurrence and reporting of ACL tibial cyst is low. Thus, every piece of information will contribute to better understanding of incidence, natural history, pathology, and best possible management of tibial cysts after ACLR.

The strength of this scoping review is that the authors have managed to create an up-to-date evidence-based resource on tibial cysts after ACLR. Though the level of evidence is low, all the evidence consolidated will certainly help the authors of future studies to better understand the patient characteristics, preoperative imaging findings, surgical findings and biopsy related to the tibial cysts after ACLR. The resource will also facilitate clinicians who encounter this complication to be equipped with evidence-based knowledge related to tibial cysts after ACLR.

## Conclusions

In our understanding, the major finding of this scope is that tibial cyst in ACLR, is more frequently related to bio-absorbable implants, however it also has been related to other causes. The natural history behind the development of these cysts and their best possible management is still controversial. More standardised reporting on patients who develop tibial cysts is needed to further add to the existing knowledge and understanding related to the tibial cysts after ACLR in the published literature.

## Supplementary Information

**Additional file 1.** Quality assessment of included articles using the Methodological Index for Non-Randomized Studies (MINORS).

## Abbreviations

ALCR: Anterior Cruciate Ligament Reconstruction
PRISMA: Preferred Reporting Items for Systematic Reviews and Meta-analyses
PRISMA-ScR: PRISMA extension for scoping reviews
ACL: Anterior cruciate ligament
MRI: Magnetic resonance imaging
MINORS: Methodological Index for Non-Randomized Studies
GRADE: Grading of Recommendations Assessment, Development and Evaluation)
PLLA: Poly(L-lactic) acid
HA: Hydroxyapatite
B-TCP: B-tricalcium phosphate
