# Supplementary material for: A comprehensive scoping review of tibial cysts after anterior cruciate ligament reconstruction
Source: J Exp Orthop. 2021 Jun 21;8:40. doi: 10.1186/s40634-021-00356-9 (PMC8215013; doi:10.1186/s40634-021-00356-9)
Supplement: Supplementary file 1 — Additional file 1. Quality assessment of included articles using the Methodological Index for Non-Randomized Studies (MINORS). [file 40634_2021_356_MOESM1_ESM.docx]

**Additional file 1** Quality assessment of included articles using the Methodological Index for Non-Randomized Studies (MINORS).

| **Author, Year** | **Herrera**  **2014** | **Chevallier**  **2018** | **Zicaro**  **206** | **Ramsingh**  **2014** | **Lomas**  **2011** | **Deie**  **2000** | **Bourke**  **2013** | **Gaweda**  **2009** |
| --- | --- | --- | --- | --- | --- | --- | --- | --- |
| **LEVEL OF EVIDENCE / STUDY DESIGN** | IV/Case series | IV/Case series | IV/Case series | IV/Case series | IV/Case series | IV/Case series | III/Nonrandom ized trial | II/Rand omized trial |
| **A CLEARLY STATED AIM** | 2 | 2 | 2 | 2 | 1 | 1 | 2 | 2 |
| **INCLUSION OF**  **CONSECUTIVE PATIENTS** | 2 | 2 | 2 | 2 | 2 | 2 | 2 | 2 |
| **PROSPECTIVE COLLECTION OF DATA** | 0 | 0 | 0 | 0 | 0 | 2 | 2 | 1 |
| **ENDPOINTS**  **APPROPRIATE TO THE AIM OF THE STUDY** | 0 | 2 | 0 | 0 | 0 | 0 | 0 | 0 |
| **UNBIASED**  **ASSESSMENT OF THE STUDY ENDPOINT** | 0 | 0 | 0 | 0 | 0 | 0 | 2 | 0 |
| **FOLLOW-UP PERIOD**  **APPROPRIATE TO THE AIM OF THE STUDY** | 2 | 2 | 1 | 1 | 1 | 1 | 1 | 1 |
| **LOSS TO FOLLOW UP LESS THAN 5%** | 2 | 2 | 2 | 2 | 1 | 2 | 1 | 0 |
| **PROSPECTIVE**  **CALCULATION OF THE STUDY SIZE** | 0 | 0 | 0 | 0 | 0 | 0 | 1 | 0 |
| **AN ADEQUATE CONTROL GROUP** | NA | NA | NA | NA | NA | NA | 2 | 2 |
| **CONTEMPORARY GROUPS** | NA | NA | NA | NA | NA | NA | 2 | 2 |
